# Sociodemographic disparities in incidence rates of advanced and low-risk prostate cancer as a proxy for diagnostic activity

**DOI:** 10.2340/1651-226X.2025.43399

**Published:** 2025-05-15

**Authors:** Ulf Strömberg, Carl Bonander, Hans Garmo, Mats Lambe, Pär Stattin, Ola Bratt

**Affiliations:** a School of Public Health and Community Medicine, Institute of Medicine, Sahlgrenska Academy, University of Gothenburg, Sweden; b Regional Cancer Centre Mid-Sweden, Uppsala University Hospital, Uppsala, Sweden; c Department of Surgical Sciences, Uppsala University, Uppsala, Sweden; d Department of Medical Epidemiology and Biostatistics, Karolinska Institute, Stockholm, Sweden; e Department of Urology, Institute of Clinical Sciences, Sahlgrenska Academy, University of Gothenburg, Sweden; f Department of Urology, Sahlgrenska University Hospital, Gothenburg, Sweden

**Keywords:** Prostate cancer, Early diagnosis, Socioeconomic factors, Ethnicity, Incidence study

## Abstract

**Background:**

Inequity in prostate cancer detection can be assessed by relating the diagnostic intensity to the incidence rate of advanced disease in different population groups, according to factors such as socioeconomic status or ethnicity.

**Methods:**

We used nationwide Swedish register data from Prostate Cancer data Base Sweden 5.0 and Statistics Sweden, which enabled us to estimate incidence rates of low-risk prostate cancer (a proxy for diagnostic activity) and advanced disease (locally advanced and/or metastatic) across population groups according to household income, country of birth, and neighborhood-level characteristics.

**Results:**

We found a gradient in the age-standardized incidence of low-risk prostate cancer across income groups, from 60 per 100,000/year in men with high to 34 per 100,000/year in men with low household income: adjusted incidence rate ratio (IRR) 0.65 (95% confidence interval [CI] 0.59–0.71). The gradient in the incidence of advanced disease had the opposite direction, from 44 to 60 per 100,000/year, IRR 1.43 (95% CI 1.31–1.56). Immigrants from a non-Nordic country (nearly 40% from Asia) had lower incidence rates of both low-risk (IRR 0.47, 95% CI 0.42–0.53) and advanced disease (IRR 0.65, 95% CI 0.58–0.73) than men born in a Nordic country. Neighborhood-level analysis considering economic standard, share of immigrants, and degree of urbanization did not clearly differentiate the incidence of advanced disease.

**Interpretation:**

Our results suggest that measures to facilitate early detection of prostate cancer should be targeted to men with a low income. A low diagnostic activity for prostate cancer among immigrants from countries with low background risk may not imply unjustified social disparity.

## Introduction

Early detection is key for successful cancer treatment, so disparities leading to that cancer is diagnosed in late stages may be particularly detrimental for socioeconomically vulnerable groups. Several studies have found that men with short education or income are more likely to be diagnosed with advanced prostate cancer [1–6]. Prostate cancer is often too advanced to be cured after symptoms occur. Greater diagnostic activity for prostate cancer, usually initiated by serum prostate-specific antigen (PSA) testing, is associated with lower incidence of advanced disease at diagnosis [7]. Screening with PSA as the primary test is therefore a way to reduce prostate cancer mortality. Organized PSA-based screening has proven to reduce prostate cancer mortality [8]. However, since screening also results in substantial overdiagnosis of slow-growing, localized cancers [8], national healthcare authorities do not recommend population-based screening [9]. Unorganized PSA testing is less effective and results in a less favorable benefit-to-harm ratio than organized screening [10] but is nonetheless widespread in many countries [9].

The great variation in background risk of prostate cancer across ethnic groups [11] complicates epidemiological surveillance of sociodemographic disparity in prostate cancer detection and care. In the US population, for example, Black men develop prostate cancer earlier in life, have more aggressive disease and higher mortality than White men, and may benefit from targeted screening approaches [12]. Policy implications may be different for other ethnic groups.

Inequity in prostate cancer detection can be assessed by relating a measure of the diagnostic activity to the incidence rate of advanced disease in different population groups, according to sociodemographic factors. Sweden’s detailed, high-quality, national registers are well suited for such assessments. We used Swedish register data on both individual- and neighborhood-level to explore inequities in prostate cancer detection by relating the incidence rate of low-risk disease as a proxy for the diagnostic activity (PSA testing and subsequent examinations) to the incidence rate of advanced disease (including locally advanced and metastatic cancer at diagnosis). We hypothesized that low income is associated with low diagnostic activity for prostate cancer and, consequently, a high incidence of advanced disease, and that these associations vary by country of birth.

## Methods

### Data on incident prostate cancer cases (numerator data)

The National Prostate Cancer Register (NPCR) of Sweden is a clinical cancer register with the aim to improve quality of care for men with prostate cancer foremost by assessing adherence to national guidelines [13, 14]. NPCR data have high validity and are 98% complete compared with the National Cancer Register [15], to which reporting of all incident cancers is mandated by law. In Prostate Cancer data Base Sweden (PCBaSe), version 5.0, NPCR has been merged with data from several other national health care registers and demographic databases by use of the unique personal identification number assigned to all permanent residents of Sweden [16].

We retrieved data on men diagnosed with prostate cancer cases during 2018–2019 as registered in NPCR. Data for men diagnosed in 2020 were available in PCBaSe 5.0, but we chose not to include them because the transient decline during the COVID-19 pandemic might have affected reporting differently across socioeconomic population strata. Based on the NPCR data on prostate cancer characteristics at diagnosis, we categorized the disease as low-risk (local stage T1-2 and Nx/N0 and M0 and Gleason score 6 and PSA < 10 ng/ml) or locally advanced or metastatic (T3-4 and/or N1 and/or M1 and/or PSA ≥ 100 ng/ml; named ‘advanced disease’ for brevity). Each case was categorized according to his year of diagnosis (2018, 2019) and age group at diagnosis (40–44, 45–49, …, 80–89, 90+).

Individual-level sociodemographic data were obtained from Statistics Sweden. We used data on *household income*, defined as the disposable income per household per consumption unit, and *country of birth*. Household income was calculated for the calendar year preceding the year of diagnosis (with a few exceptions when the income data for that year were incomplete; for such cases, complete income data for the calendar year before were used). We categorized household income as ‘low’, corresponding to the 1st quartile of the household income distribution for all residents of Sweden based on Statistic Sweden’s income data for the calendar year at issue, ‘intermediate’ (2nd and 3rd quartiles) or ‘high’ (4th quartile). Country of birth was categorized as Nordic, EU (except Sweden, Denmark, and Finland), Asia, Africa, and elsewhere.

We also considered neighborhood-level data assessed by (1) geo-coding each case to his residential neighborhood at the time of diagnosis and (2) categorizing each neighborhood according to population data provided by Statistics Sweden (the actuality of the population data was the end of the year of diagnosis, 2018 or 2019; see Population data). The neighborhoods correspond to Statistics Sweden’s small-area division of Sweden referred to as Demographic Statistical Areas, launched in 2018, to facilitate the monitoring of segregation and socioeconomic conditions [17]. In 2018, the populations across the 5,984 Demographic Statistical Areas in Sweden – referred to as the neighborhoods in the following – varied between 650 and 4,250 individuals, with a median of 1,600 people.

Specifically, we considered three neighborhood-level covariates: (1) *economic standard*, (2) *proportion of inhabitants born outside the Nordic countries*, and (3) *grade of urbanization*. Economic standard was defined as the proportion of inhabitants with low household income out of all inhabitants of a neighborhood. Covariates 1 and 2 were categorized into national quintiles Q1–Q5 (Q1 = 20% of the 5,984 neighborhoods in Sweden with the lowest proportions (for economic standard, meaning the highest standard), and so on). Economic standard has been suggested as an appropriate neighborhood-level deprivation indicator for Sweden [18]. The second covariate, proportion with a non-Nordic country of birth, should be kept distinct because the impact on health of ethnic composition or geographical origins of a local population could be different from that of material deprivation [18, 19]. The third covariate, grade of urbanization, was categorized into rural, semi-urban, and urban [17]. Eighteen percent of the 5,984 neighborhoods are classified as rural, 10% as semi-urban, and 72% as urban.

### Population data (denominator data)

Counts for the entire Swedish population were obtained from Statistics Sweden, as registered at the end of each calendar year under study (2018, 2019), by neighborhood, sex, 5-year age groups (older than 89 years was grouped as 90+), household income (low, intermediate, and high), and country of birth (Nordic, non-Nordic).

The available population data enabled matching denominators to numerators (groups of cases) according to the specified variables. We decided to use population data at the end of each observation year because (1) the neighborhood-level sociodemographic variables (economic standard and proportion with a non-Nordic country of birth) were assessed for the inhabitants registered in each neighborhood at the end of each calendar year and (2) our preference of analogous population data (denominator data) for the individual- and neighborhood-level analyses. The total number of men aged 40 years or older registered in Sweden increased only marginally between the end of 2017 and the end of 2019 (2% increase).

For the finer grouping of country of birth into Nordic, EU (except Nordic), Asia, Africa, and elsewhere – in accordance with the case data – we retrieved corresponding population data by 5-year age groups only. Hence, the finer grouping of birth country was used only for calculations of age-standardized incidence rates.

### Statistical methods

For descriptive purposes, we calculated the age-standardized incidence rates of all prostate cancers, low-risk disease, and advanced disease – in each year and in each defined population group – using the European Standard Population revision 2013, that is, the following weights across the age groups 40–44, 45–49, …, 85–89, and 90+ years: 0.070, 0.070, 0.070, 0.065, 0.060, 0.055, 0.050, 0.040, 0.025, 0.015, and 0.010. The presented age-standardized incidence rates refer to the total male population (all ages) in Sweden, although the age-specific rates up to 40 years are practically zero. Using Byar’s method [20], we calculated a 95% confidence interval (CI) around each age-standardized incidence rate.

Poisson regression was employed (IBM SPSS Statistics version 29.0) to estimate associations between the individual-level covariates (household income and country of birth) and age-adjusted (and calendar year adjusted) incidence rates of low-risk prostate cancer and advanced disease, respectively. Case data were aggregated for each covariate, and the corresponding denominators were log-transformed and used as offsets. We report incidence rate ratios (IRRs) with 95% CIs.

For the neighborhood-level analysis, we employed Bayesian models where spatially structured random effects were modeled using an intrinsic conditional autoregressive prior [21]. Such a Bayesian model provides both local and global smoothing on the underlying incidence rate in each neighborhood. Thereby, robust age-adjusted (and calendar year adjusted) estimates of the underlying incidence rates are obtained [21]. By incorporating fixed-effect parameters for the neighborhood-level covariates, we estimated the associations of each covariate on the stage-specific incidence rates. We report IRRs with 95% CIs. The Supplementary Methods contains a detailed description of the Bayesian modeling approach.

## Results

The total population of men aged 40 years or older registered in Sweden was 2,537,838 at the end of 2018 and 2,564,153 at the end of 2019. We obtained data from PCBaSe on 21,874 men diagnosed with prostate cancer at the age of 40 years or older in these 2 years, of whom at the time of diagnosis, 5,019 (22.9%) had low-risk disease and 4,881 (22.3%) had advanced disease. Only seven cases were diagnosed at an age below 40 years.

The annual overall age-standardized incidence rates of prostate cancer were estimated to 225 (95% CI 220–229) per 100,000 men in 2018 and 221 (95% CI 216–225) per 100,000 men in 2019 (Table 1).

**Table 1:** Number of incident prostate cancer cases (all types, low-risk, and locally advanced or metastatic) and the age-standardized incidence rates by the calendar year and the individual-level variables *household income* and *country of birth*.

Men aged ≥ 40 years registered in Sweden 2018/2019 divided by	All types of prostate cancer		Low-risk prostate cancer		Locally advanced or metastatic prostate cancer	
	Number of cases	Age-standardized^a^ incidence rate per 100,000/year (95% CI)	Number of cases (% of all types of prostate cancer cases)	Age-standardized^a^ incidence rate per 100,000/year (95% CI)	Number of cases (% of all types of prostate cancer cases)	Age-standardized^a^ incidence rate per 100,000/year (95% CI)
Calendar year						
2018	10,943	225 (220–229)	2,588 (23.6)	53 (51–55)	2,477 (22.6)	52 (50–54)
2019	10,931	221 (216–225)	2,431 (22.2)	49 (47–51)	2,404 (22.0)	49 (47–51)
Household income						
High	7,173	238 (232–245)	2,130 (29.7)	60 (57–63)	1,020 (14.2)	44 (41–48)
Intermediate	10,489	218 (214–222)	2,246 (21.4)	49 (47–51)	2,428 (23.1)	49 (47–52)
Low	4,212	193 (187–199)	643 (15.2)	34 (31–37)	1,433 (34.0)	60 (57–63)
Country of birth						
Nordic	20,512	234 (230–237)	4,738 (23.1)	55 (53–56)	4,563 (22.2)	52 (51–54)
Non-Nordic^b^	1,362	139 (131–147)	281 (20.6)	24 (21–27)	318 (23.3)	39 (34–44)
- EU (except Nordic)	478	159 (145–174)	88 (18.4)	30 (24–36)	125 (26.2)	42 (35–50)
- Asia	312	106 (91–121)	78 (25.0)	21 (16–28)	69 (22.1)	32 (23–43)
- Africa^c^	126	172 (132–218)	31 (24.6)	26 (16–39)	28 (22.2)	53 (26–88)
- Elsewhere	446	139 (126–154)	84 (18.8)	22 (17–27)	96 (21.5)	37 (29–46)

CI: confidence interval.

a Referring to the total male population (all ages), according to the European Standard Population revision 2013 (see Statistical methods).

b In the Swedish population of men aged 40 years or older (in 2018/2019) *and* born in a non-Nordic country (16% of the total study population), 23% were born in the EU, 37% in Asia, 11% in Africa, and 29% elsewhere.

c Among the men from Africa, 25% were born in North Africa, where the background risk of prostate cancer is low [11]. The other part of Africa is to large extent a high-risk area [11].

With the finer grouping of immigrant groups, we generally obtained lower age-standardized incidence rates – most notably for the group from Asia comprising 37% of the non-Nordic immigrants – compared with the Nordic-born men (Table 1). The only exception was the marginally higher age-standardized incidence rate of advanced prostate cancer for men from Africa.

### Individual-level analysis

**Figure 1:**
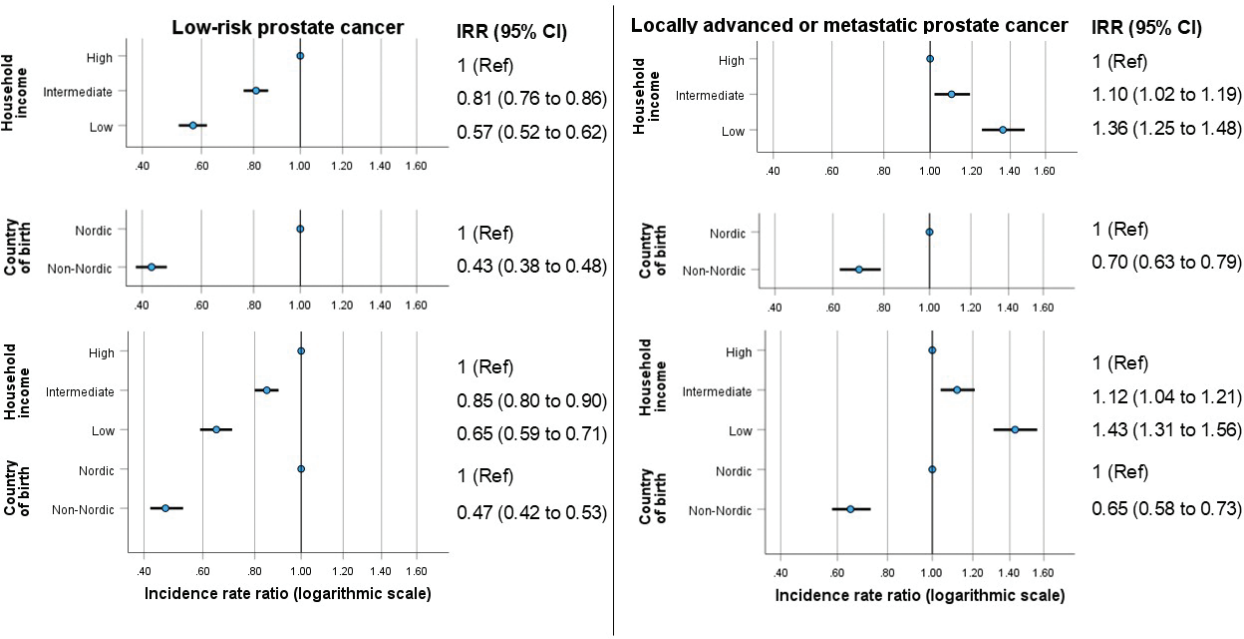
Associations of the individual-level covariates *household income* and *country of birth* on the incidences rates of low-risk (panels on the left) and locally advanced or metastatic (panels on the right) prostate cancer at the time of diagnosis in Sweden, 2018–2019. The upper four panels show the age-adjusted incidence rate ratios (IRRs) (horizontal bars indicate 95% confidence intervals) obtained from the single covariate Poisson models, and the lower two panels show the age- and covariate-adjusted estimates from the models with both covariates included.

The age-standardized incidence rate of low-risk prostate cancer was lower among men with a low household income (34 per 100,000/year) and in men born in a non-Nordic country (24 per 100,000/year) than in men with intermediate or high household income or born in a Nordic country (e.g. 60 per 100,000/year in men with a high household income, Table 1). For advanced disease, the incidence gradient was reversed and increased across the groups of men categorized by high, intermediate, and low household income (from 44 to 60 per 100,000/year) but, just as for low-risk prostate cancer, the incidence among men born in a non-Nordic country was lower than in those born in a Nordic country (39 vs. 52 per 100,000/year). The Poisson regression models, both those with (1) a single covariate and adjustment for age group and (2) the other covariate added, showed corresponding statistically significant associations (Figure 1). For example, the models with both covariates included yielded an IRR for men with low versus high household income of 0.65 (95% CI 0.59–0.71) for low-risk prostate cancer; the corresponding IRR was 1.43 (95% CI 1.31–1.56) for advanced disease.

### Neighborhood-level analysis

Ten cases that could not be geo-coded because of a protected or unknown address were excluded from the neighborhood-level analysis. In the population of men aged 40 years or older registered in Sweden, 5,557 men with a protected/unknown address were excluded from the total denominator for the year 2018, and 5,862 similarly for year 2019.

The age-standardized incidence rate of low-risk prostate cancer was lower among men living in neighborhoods with a lower economic standard (range: from 56 to 42 per 100,000/year) or a higher proportion of residents born in a non-Nordic country (range: from 58 to 36 per 100,000/year) (Table 2). There was also a declining, but less pronounced, gradient for the age-standardized incidence of advanced disease with the neighborhoods’ proportion of residents born in a non-Nordic country (from 58 to 48 per 100,000/year), while no clear gradient was observed on the neighborhoods’ economic standard (Table 2). Men living in urban areas had lower age-standardized incidence rates of both low-risk and advanced disease than men living in rural areas (48 vs. 58 and 49 vs. 57 per 100,000/year, respectively, Table 2).

**Table 2:** Number of incident prostate cancer cases (all types, low-risk, and locally advanced or metastatic) and the age-standardized incidence rates by the neighborhood-level covariates *economic standard, proportion with a non-Nordic country of birth*, and *grade of urbanization*.

Men aged ≥ 40 years registered in Sweden 2018/2019 divided by	All types of prostate cancer		Low-risk prostate cancer		Locally advanced or metastatic prostate cancer	
	Number of cases	Age-standardized^a^ incidence rate per 100,000/year (95% CI)	Number of cases (% of all types of prostate cancer cases)	Age-standardized^a^ incidence rate per 100,000/year (95% CI)	Number of cases (% of all types of prostate cancer cases)	Age-standardized^a^ incidence rate per 100,000/year (95% CI)
Economic standard (national quintiles)						
Q1 (highest)	4,575	244 (237–252)	1,080 (23.6)	56 (52–59)	854 (18.7)	49 (46–53)
Q2	4,732	232 (225–239)	1,101 (23.3)	53 (50–56)	999 (21.1)	51 (49–54)
Q3	4,523	220 (214–227)	1,059 (23.4)	52 (49–55)	1,008 (22.3)	50 (47–53)
Q4	4,578	220 (213–226)	1,043 (22.8)	51 (48–54)	1,125 (24.6)	54 (51–57)
Q5 (lowest)	3,449	195 (189–202)	730 (21.2)	42 (39–45)	891 (25.8)	51 (47–54)
Proportion with a non-Nordic country of birth (national quintiles)						
Q1 (lowest)	5,272	241 (234–248)	1,276 (24.2)	58 (55–61)	1,210 (23.0)	58 (54–61)
Q2	5,081	238 (231–245)	1,213 (23.9)	57 (54–61)	1,097 (21.6)	52 (49–55)
Q3	4,587	231 (225–238)	1,033 (22.5)	52 (49–56)	928 (20.2)	47 (44–50)
Q4	3,984	211 (204–217)	899 (22.6)	48 (45–51)	910 (22.8)	49 (46–52)
Q5 (highest)	2,933	184 (177–191)	592 (20.2)	36 (33–39)	732 (25.0)	48 (44–51)
Grade of urbanization						
Rural	4,917	236 (229–242)	1,241 (25.2)	58 (55–61)	1,113 (22.6)	57 (53–60)
Semi-urban	2,139	224 (215–234)	497 (23.2)	54 (49–59)	492 (23.0)	51 (47–56)
Urban	14,801	219 (215–222)	3,275 (22.1)	48 (47–50)	3,272 (22.1)	49 (48–51)

CI, confidence interval.

a Referring to the total male population (all ages), according to the European Standard Population revision 2013 (see Statistical methods).

The single covariate models yielded statistically significant associations between each covariate and the age-adjusted incidence of low-risk prostate cancer (Table 3): the IRRs were 0.68 (95% CI 0.61–0.71) for men residing in neighborhoods with lowest versus highest economic standard, 0.70 (95% CI 0.63–0.79) for men residing in neighborhoods with highest versus lowest proportion of residents born in a non-Nordic country, and 0.88 (95% CI 0.82–0.96) for men living in urban versus rural areas. The associations with economic standard and degree of urbanization were only marginally changed by including all three covariates in the model, whereas the association with the proportion of residents born in a non-Nordic country disappeared. Associations on the age-adjusted incidence rates of advanced disease were either absent or less pronounced (Table 3); in the fully adjusted models, only Q3 for the proportion of non-Nordic immigrants had a 95% CI not crossing 1.

**Table 3:** Associations of the neighborhood-level covariates *economic standard*, *proportion with a non-Nordic country of birth*, and *grade of urbanization* on the age-adjusted incidence rates of low-risk and locally advanced or metastatic prostate cancer at the time of diagnosis in Sweden, 2018–2019.

Covariate	Low-risk prostate cancer		Locally advanced or metastatic prostate cancer	
	IRR (95% CI)^a^	Additional covariate-adjusted IRR (95% CI)^b^	IRR (95% CI)^a^	Additional covariate-adjusted IRR (95% CI)^b^
Economic standard (national quintiles)				
Q1 (highest)	1 (Ref)	1 (Ref)	1 (Ref)	1 (Ref)
Q2	0.93 (0.85–1.01)	0.92 (0.83–1.00)	1.04 (0.95–1.15)	1.04 (0.95–1.15)
Q3	0.82 (0.75–0.90)	0.81 (0.73–0.90)	0.96 (0.87–1.05)	0.97 (0.87–1.07)
Q4	0.80 (0.73–0.88)	0.79 (0.71–0.89)	1.01 (0.91–1.11)	1.05 (0.93–1.17)
Q5 (lowest)	0.68 (0.61–0.75)	0.72 (0.62–0.83)	0.93 (0.84–1.03)	0.99 (0.86–1.14)
Proportion with a non-Nordic country of birth (national quintiles)				
Q1 (lowest)	1 (Ref)	1 (Ref)	1 (Ref)	1 (Ref)
Q2	0.99 (0.91–1.08)	1.07 (0.97–1.17)	0.92 (0.85–1.00)	0.93 (0.85–1.02)
Q3	0.97 (0.88–1.06)	1.10 (0.99–1.23)	0.87 (0.80–0.96)	0.89 (0.79–0.99)
Q4	0.89 (0.80–0.98)	1.08 (0.95–1.23)	0.89 (0.81–0.98)	0.91 (0.80–1.03)
Q5 (highest)	0.70 (0.63–0.79)	0.95 (0.80–1.13)	0.86 (0.78–0.96)	0.87 (0.76–1.04)
Grade of urbanization				
Rural	1 (Ref)	1 (Ref)	1 (Ref)	1 (Ref)
Semi-urban	0.92 (0.82–1.03)	0.92 (0.82–1.03)	0.90 (0.81–1.00)	0.93 (0.83–1.04)
Urban	0.88 (0.82–0.96)	0.89 (0.82–0.98)	0.91 (0.84–0.98)	0.96 (0.88–1.05)

IRR: incidence rate ratio; CI: confidence interval; Q1-Q5: quintiles.

a Age-adjusted IRR estimates obtained from the employed model (see Statistical methods) with a single covariate included.

b Age- and covariate-adjusted IRR estimates obtained from the employed model (see Statistical methods) with the three covariates included.

The Bayesian modeling indicated substantial residual spatial correlations in the incidence patterns that cannot be explained by the neighborhood-level characteristics we included in our models (Supplementary Table S1).

## Discussion

In Sweden, the age-standardized incidence rate of low-risk prostate cancer, a marker of diagnostic activity in general and PSA testing in particular, was lower in men with low household income compared with high household income and in those born in a non-Nordic country compared with Nordic-born men. The observed age-standardized incidence rate of advanced disease was, as expected, reciprocally higher in men with low household income compared with a high household income. In contrast, despite evidence for a low diagnostic activity in men born in a non-Nordic country, their incidence of advanced disease was also low.

We are unaware of any previously published studies that have used a measure of diagnostic activity to assess to what extent sociodemographic disparities in the incidence rates of advanced prostate cancer depend on inequalities in the diagnostic activity for early detection. Commonly, studies on sociodemographic variations in tumor stage at diagnosis are based on data for diagnosed cases only. Only a few studies have incorporated population (denominator) data to evaluate stage-specific incidence patterns for prostate cancer. A French regional study reported that the total incidence rate of prostate cancer in 2006 to 2010 was lower, but the incidence of intermediate- to high-grade disease and prostate cancer mortality was higher in the most disadvantaged areas [4]. In a US-based study, the investigators found a higher incidence of advanced prostate cancer in men younger than 75 years in counties with higher poverty levels, but they did not relate this finding to the incidence of localized disease [2]. In a recently published study from England, the investigators found that greater socioeconomic deprivation was linked to higher metastatic but lower overall prostate cancer incidence [5].

Low-risk prostate cancer is typically diagnosed after an initial PSA test and a systematic prostate biopsy [6]. Reasonably, in each population group, the incidence rate of low-risk prostate cancer can serve as a surrogate marker of the diagnostic activity related to PSA testing. One should bear in mind, though, that some subpopulations have a lower background risk, implying that it may be inadequate to consider a low diagnostic inactivity in these subpopulations as an unjustified disparity. This limitation shows the importance of considering the measure of the diagnostic activity (in our study the incidence rate of low-risk prostate cancer) in relation to the incidence rate of advanced disease. It is worth noticing that low-risk prostate cancer is indolent or at least very slow-growing and, when detected, usually represents overdiagnosis. This means that the lower incidence of advanced prostate cancer in men with high income comes at the price of more overdiagnosis.

Our findings of a lower incidence of low-risk prostate cancer and a higher incidence of advanced disease in men with lower income were expected and agree with previous reports [2, 4, 5, 22–24]. Indeed, results from the Swedish Gothenburg-1 prostate cancer screening trial suggest that the benefit of an organized screening program is greater among men with a low educational level, which is strongly associated with a low income in many countries (including Sweden [25]) than among men with a higher educational level [26].

We have recently reported results, suggesting that socioeconomic disparity affects the diagnostic part of the prostate cancer pathway more than subsequent management in specialist care in Sweden: low educational level as well as low income was associated with cancer-specific mortality, but after adjustment for cancer characteristics, the associations were attenuated and no longer statistically significant [27].

It was not surprising that the age-standardized incidence of low-risk cancer was lower in men born in a non-Nordic versus a Nordic country. Interestingly, the incidence of advanced disease was, despite this, also lower in men born in a non-Nordic country. This is likely explained by a lower background risk of prostate cancer in the dominant South European and Asian immigrant groups in Sweden. The age-standardized incidence rate of advanced disease in men born in a non-Nordic country was even lower than in men with a high income, despite a more than twice as high age-standardized incidence of low-risk disease among the latter. These findings are in line with the results of a study of men diagnosed with prostate cancer in Sweden between 1991 and 2009 [28]. In that study, the risk of low-risk disease and the likelihood of being diagnosed after a health check-up were lower among immigrants, particularly among men from Asia, but the risk of metastatic disease was lower than in Swedish-born men.

Sweden does not allow classification of ethnicity, in contrast to, for instance, the US and the UK. We were not able to address stage-specific incidence rates in men with Black ethnicity, whereas, in England, higher incidence of both metastatic and overall cancer has been observed in neighborhoods with more men with Black ethnicity [5].

The neighborhood-level analyses showed weaker associations than the individual-level analyses. Indeed, unless contextual effects are pronounced [29], a gradient across neighborhoods sorted by an aggregate summary of an individual-level covariate is typically weaker than the corresponding gradient over an individual-level covariate. Nonetheless, statistically significant gradients of potential inequity concern were found for the incidence rate of low-risk prostate cancer by economic standard and by proportion of non-Nordic immigrants. In contrast, however, the neighborhood-level gradients for the incidence of advanced disease were weaker, not supporting any clear inequity that might be considered for targeted interventions to promote early detection. This finding can be attributed to strong correlations between economic standard, proportion of residents born in a non-Nordic country, and degree of urbanization across neighborhoods in Sweden (Supplementary Figure S1), implying that the associations between the advanced disease incidence, on the one hand, and the individual-level covariates household income and country of birth, on the other hand, counterbalance each other on the neighborhood-level.

Although the individual-level analysis in our study had better discriminatory power than the neighborhood-level analysis, the latter is the only option in many countries. Even in countries with accessible individual sociodemographic data, neighborhood-level analysis may be preferred for practical and integrity reasons. Specifically, an analysis of stage-specific incidence patterns across neighborhoods may be preferred for identifying population groups of particular concern for targeted screening efforts [30]. Our results, which showed substantial residual spatial correlation in the stage-specific incidence rates even after considering sociodemographic differences and degree of urbanization, imply that geographical differences, and not only neighborhood-level characteristics, should be considered for such targeting. Geographical aspects may generally affect prostate cancer detection and care in Scandinavia [31, 32], and other countries with sparsely populated parts.

A strength of our study was the nationwide, population-based design and the use of information from high-quality registers for both individual- and neighborhood-level assessments. A weakness was that no data were available on PSA testing, but the incidence of low-risk prostate cancer not only is a reasonable proxy but also captures diagnostic investigations for men with a raised PSA value. Furthermore, in the multivariable analysis considering both income and country of birth, the latter variables were crudely classified into the groups Nordic and non-Nordic origin of birth. Based on the available data, we could not properly investigate associations with ethnic groups.

## Conclusions

We found evidence that, in Sweden, low household income is strongly associated with a low diagnostic activity for prostate cancer and, consequently, a high incidence of advanced disease. This may guide targeted measures to facilitate early detection of prostate cancer.

Despite a lower diagnostic activity among immigrants from non-Nordic countries, the incidence of advanced prostate cancer was substantially lower in this group than in Nordic-born men. This means that a low uptake of PSA testing and screening for prostate cancer among immigrants from a country with low prostate cancer risk may not imply unjustified social disparity.

A neighborhood-level analysis considering economic standard, share of immigrants, and degree of urbanization did not yield a clear-cut differentiation of the incidence of advanced disease. Hence, such neighborhood characteristics only are probably insufficient to use as a basis for geographically targeted screening efforts. More detailed disease mapping [33] of the incidence of low-risk and advanced prostate cancer may be a better approach for identifying neighborhoods for targeted interventions.

## Supplementary Material



## Data Availability

Aggregated data underlying this article can be shared upon request to the corresponding author.
