# Supplementary material for: Sociodemographic disparities in incidence rates of advanced and low-risk prostate cancer as a proxy for diagnostic activity
Source: Acta Oncol. 2025 May 15;64:43399. doi: 10.2340/1651-226X.2025.43399 (PMC12105542; doi:10.2340/1651-226X.2025.43399)
Supplement: Supplementary file 1 [file AO-64-43399-s1.pdf]

Supplementary material has been published as submitted. It has not been copyedited, or typeset by Acta Oncologica

Supplemental material

Supplementary Methods (on page 1)

Supplementary Table S1 (on page 2)

Supplementary Figure S1 (on page 3)

Supplementary Methods

For the neighborhood level analyses, we apply a Bayesian hierarchical Poisson regression model with spatial random effects to account for spatial dependence. Specifically, we use the Besag-York-Mollié (BYM2) model proposed by Riebler et al. [1], which improves interpretability and simplifies hyperprior specification compared to the original BYM model [2]. The model is specified as follows:

$$\ln\left(\frac{y_{ijt}}{p_{ijt}}\right) = \alpha + \pi_i + \delta_j + \gamma_t + \mathbf{X}\boldsymbol{\beta}$$

where y_{ijt} is the observed number of cases in neigbourhood i = 1, 2, …, 5984; age group j = 40-44, 45-49, …, 90+; at time (calendar year) t = 2018, 2019, and p_{ijt} is the corresponding population denominator. The model includes an intercept (α), as well as age-group (δ_j) and time (γ_t) fixed effects to adjust for age and calendar year. $\mathbf{X}\boldsymbol{\beta}$ represents additional covariates and their coefficients, which we also model as fixed. Neighborhood-specific random effects, π_i, are parameterized as

$$\boldsymbol{\pi} = \frac{1}{\sqrt{\tau_\pi}}\left(\sqrt{1-\phi}\mathbf{v} + \sqrt{\phi}\mathbf{u}_*\right),$$

where τ_π is the total precision of the neighborhood effects, and $\mathbf{v}$ and $\mathbf{u}_*$ represent unstructured and spatially structured components, with the former modelled as an i.i.d. effect and the latter using an intrinsic conditional autoregressive (ICAR) structure. The parameter ϕ is a mixing parameter that can be interpreted as the proportion of the marginal variance in the neighborhood effects attributable to the spatially structured component.

We use integrated nested Laplace approximation (INLA) [3], implemented via the R-INLA package [4], to fit the model. Penalized complexity (PC) priors [5] are applied to the precision parameter τ_π, with default settings recommended for BYM2 models in R-INLA. These priors encourage sparsity in the random effects, shrinking them toward zero when the data do not support their inclusion in the model.

References

1. Riebler A, Sørbye SH, Simpson D, Rue H. An intuitive Bayesian spatial model for disease mapping that accounts for scaling. Stat Methods Med Res. 2016;25:1145–65.

2. Besag J, York J, Mollié A. Bayesian image restoration, with two applications in spatial statistics. Ann Inst Stat Math. 1991;43:1–20.

3. Rue H, Martino S, Chopin N. Approximate Bayesian inference for latent Gaussian models by using integrated nested Laplace approximations. J R Stat Soc Ser B Stat Methodol. 2009;71:319–92.
4. Lindgren F, Rue H. Bayesian Spatial Modelling with R-INLA. J Stat Softw. 2015;63.
5. Simpson D, Rue H, Riebler A, Martins TG, Sørbye SH. Penalising Model Component Complexity: A Principled, Practical Approach to Constructing Priors. Stat Sci. 2017;32:1–28.

Supplementary Table S1 Estimates of the hyperparameters for each neighbourhood level model, reflecting the total precision for the neighbourhood effects (τ_{π}) and the proportion of the marginal variance in the neighborhood effects attributable to the spatially structured component (ϕ).

**Model**<br>*Outcome event*<br>Covariate	**Precision**<br>mean (95% CI[a])	**Spatial variance proportion**<br>mean (95% CI[a])
Low-risk prostate cancer diagnosis		
Economic standard	6.52 (4.96, 8.48)	0.95 (0.81, 1.00)
Proportion of non-Nordic immigrants	6.91 (5.20, 9.08)	0.94 (0.79, 1.00)
Degree of urbanisation	6.32 (4.81, 8.23)	0.94 (0.80, 1.00)
All three covariates included	6.68 (5.05, 8.74)	0.95 (0.80, 1.00)
Advanced disease diagnosis		
Economic standard	22.40 (12.76, 37.81)	0.83 (0.49, 0.99)
Proportion of non-Nordic immigrants	24.52 (13.26, 43.34)	0.81 (0.45, 0.98)
Degree of urbanisation	23.44 (12.94, 40.65)	0.82 (0.46, 0.99)
All three covariates included	23.83 (12.99, 41.86)	0.82 (0.46, 0.99)

[a] Credible interval

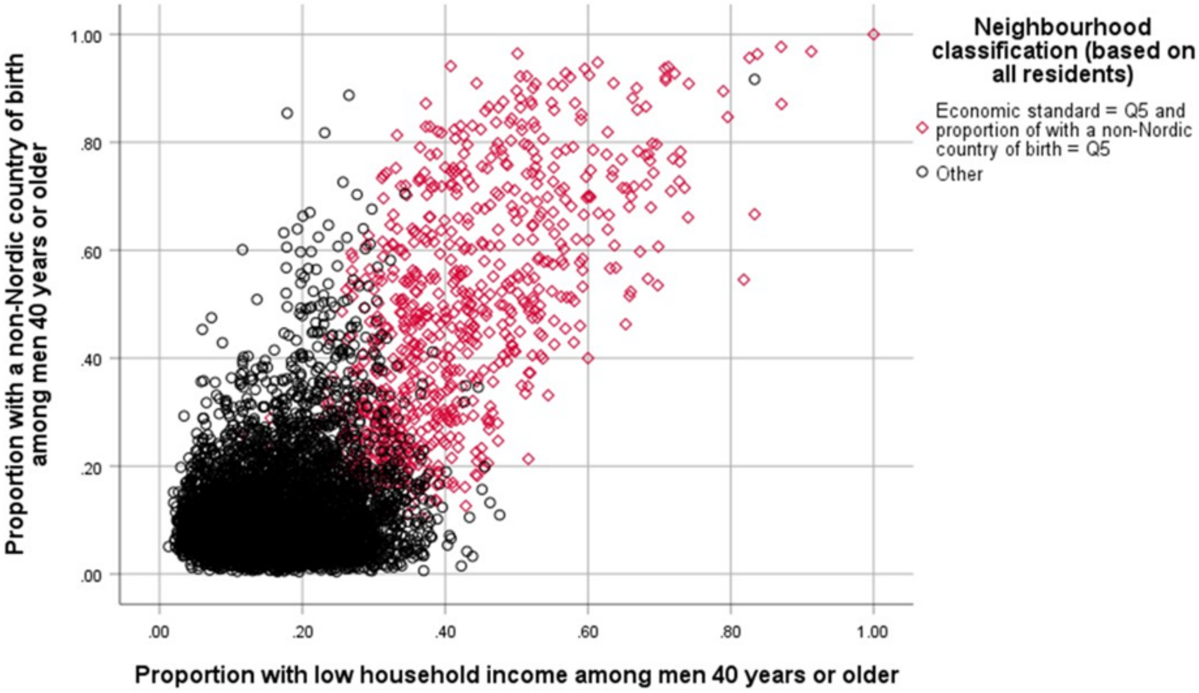

Supplementary Figure S1 Scatterplot based on the sociodemographic data on the Swedish population (year 2018) divided into 5984 neighbourhoods. The 774 neighbourhoods marked by red diamonds were classified in the upper national quintile (Q5) regarding both economic standard (proportion of residents with low household income, i.e., Q5 = lowest economic standard) and the proportion of residents born in a non-Nordic country. Of these 774 neighbourhoods, 748 was in urban areas, 23 in semi-urban areas, and 3 in rural areas. N.B.: the other single neighbourhood in the upper right part of the scatterplot was also classified in Q5 regarding economic standard and located in an urban area but classified in Q4 regarding proportion with a non-Nordic country of birth.
